# Supplementary material for: Impact of Dye Encapsulation in ZIF-8 on CO2, Water, and Wet CO2 Sorption
Source: Molecules. 2023 Oct 12;28(20):7056. doi: 10.3390/molecules28207056 (PMC10609182; doi:10.3390/molecules28207056)
Supplement: Supplementary file 1 [file molecules-28-07056-s001.zip › molecules-2609200-supplementary.pdf]

Impact of dye encapsulation in ZIF-8 on CO_2, water and wet CO_2 sorption

Aljaž Škrjanc [1,2], Mojca Opresnik [1], Matej Gabrijelčič [1,3], Andraž Šuligoj [1,4], Gregor Mali [1,2,†] and Nataša Zabukovec Logar [1,2,*]

1 Department of Inorganic Chemistry and Technology, National Institute of Chemistry, Hajdrihova 19, SI-1001 Ljubljana, Slovenia; aljaz.skrjanc@ki.si; mojca.opresnik@ki.si; matej.gabrijelcic@ki.si; andraz.suligoj@ki.si; gregor.mali@ki.si
2 University of Nova Gorica, Vipavska 13, SI-5000 Nova Gorica, Slovenia
3 Faculty of Mathematics and Physics, University of Ljubljana, Jadranska ulica 19, SI-1000 Ljubljana
4 Faculty of Chemistry and Chemical Technology, University of Ljubjana, Večna pot 113, SI-1000 Ljubljana
† Deceased
* Correspondence: natasa.zabukovec@ki.si

Supporting information

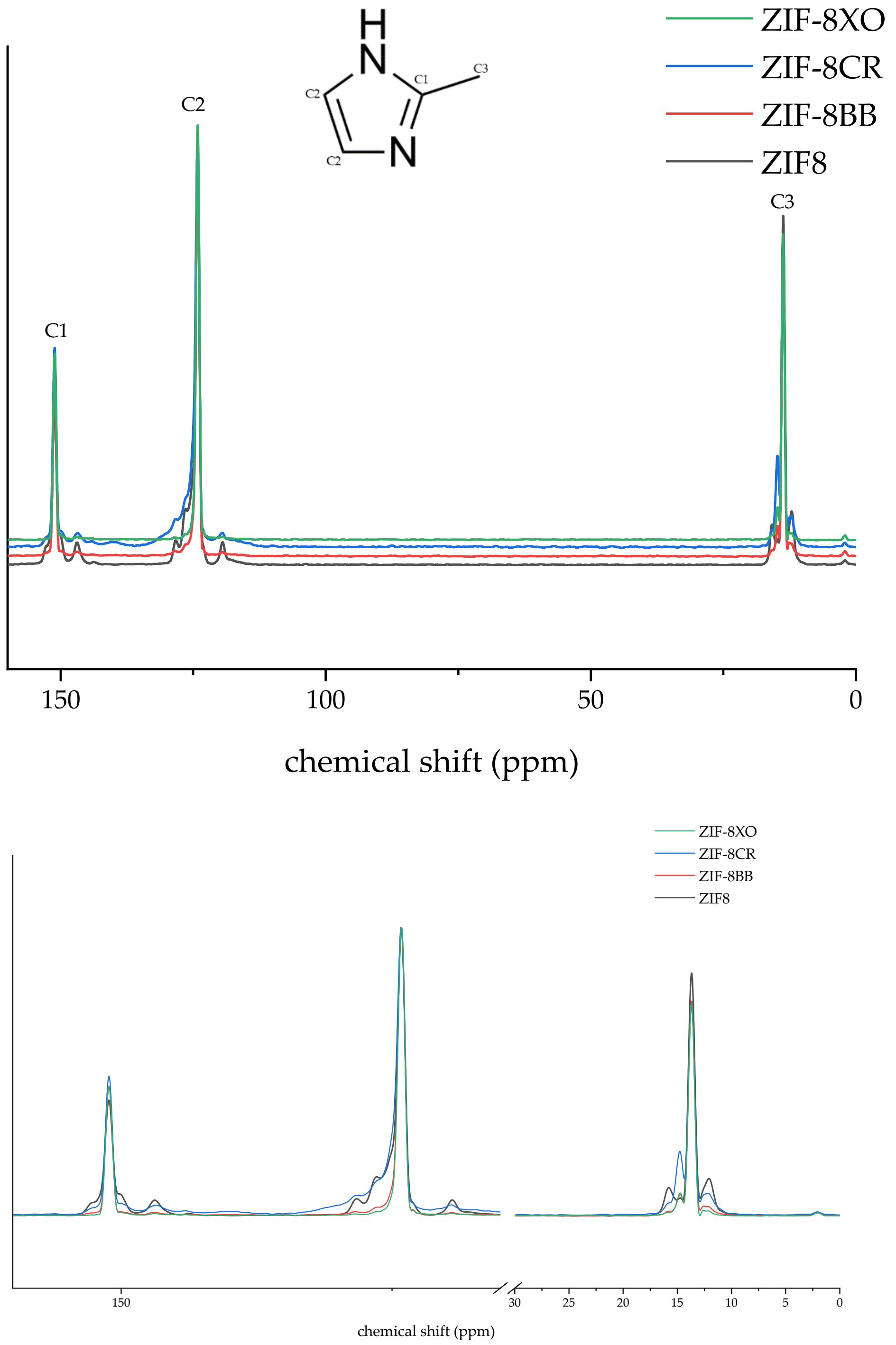

Figure S1: ^1H-^{13}C CPMAS spectra of parent ZIF-8 and ZIF-8OD samples.

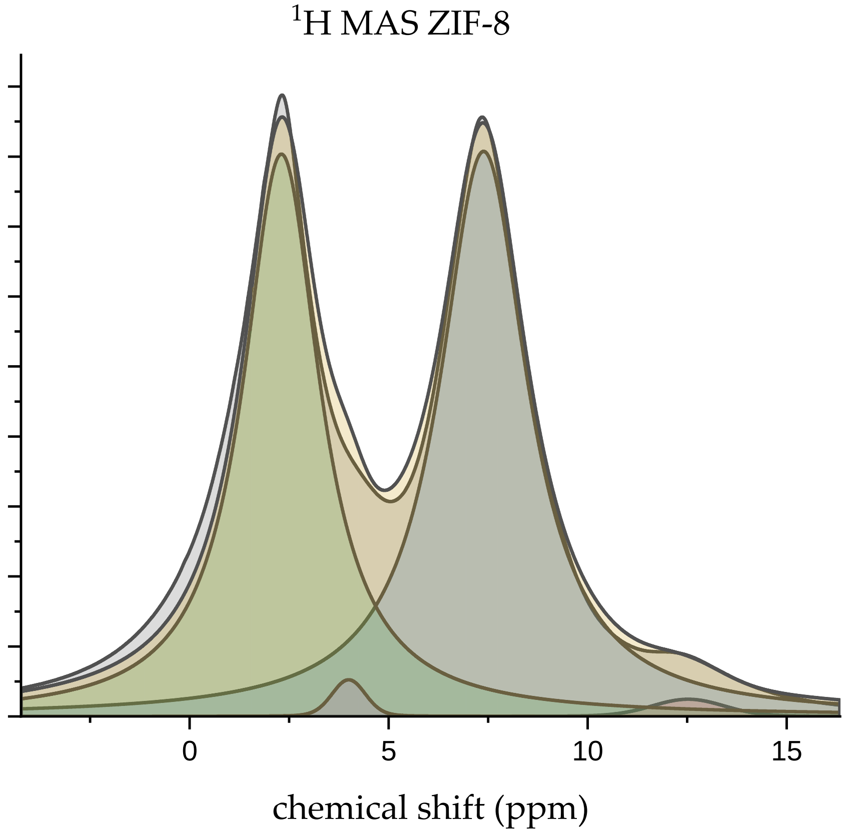

[1]H MAS ZIF-8
0
5
10
15
chemical shift (ppm)

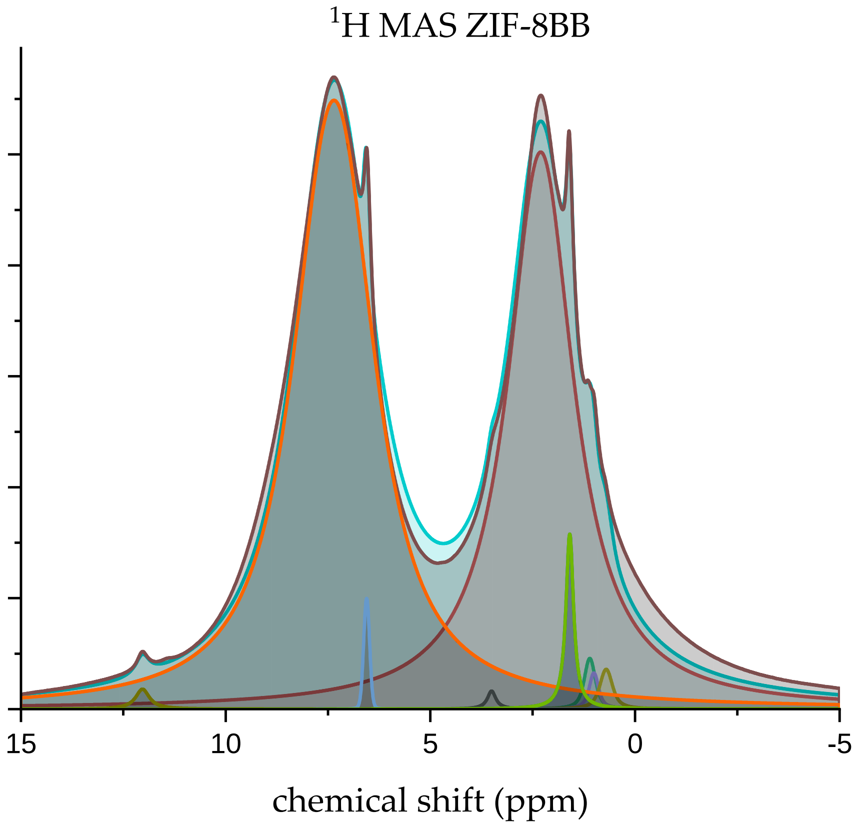

[1]H MAS ZIF-8BB
15
10
5
0
-5
chemical shift (ppm)

1H MAS ZIF-8XO

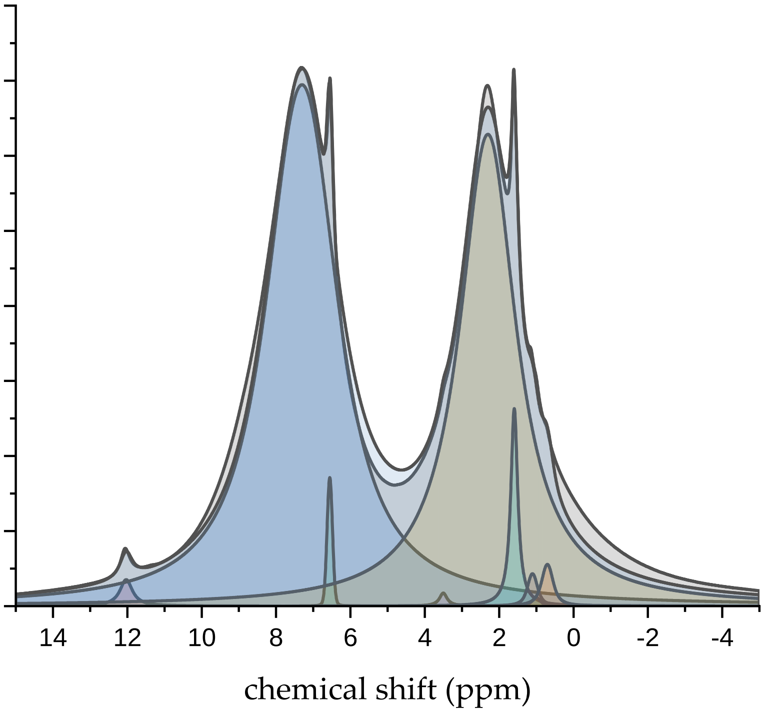

1H MAS ZIF-8CR

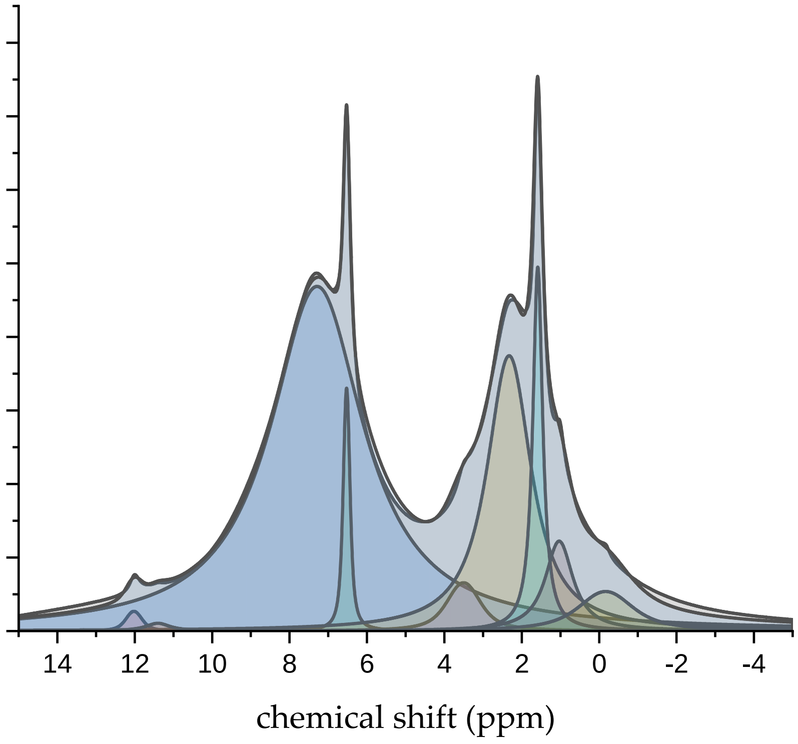

Figure S2: ^{1}H MAS NMR deconvoluted spectra of parent ZIF-8 and ZIF-8OD samples.

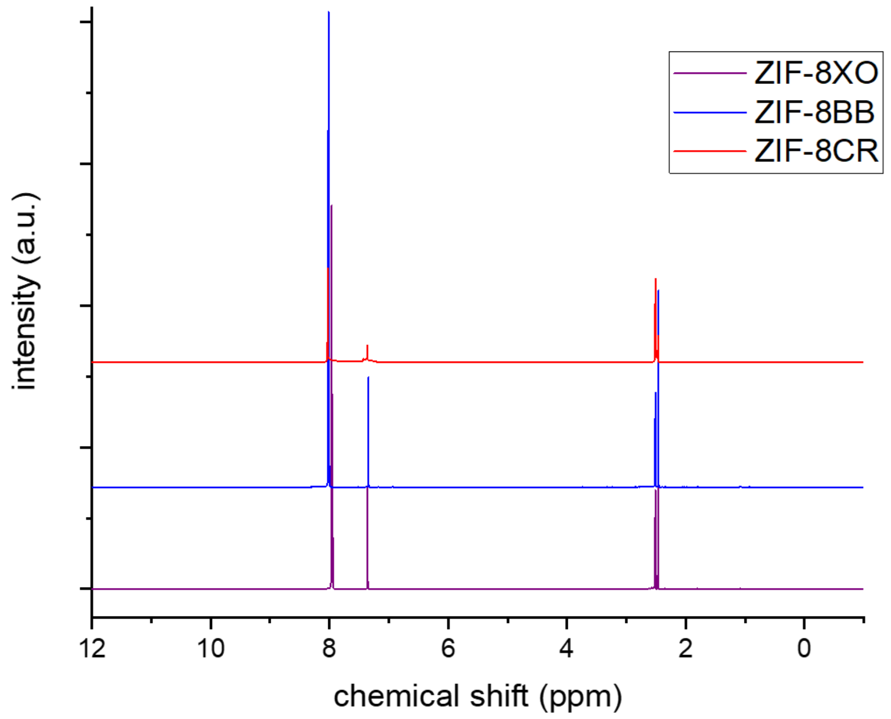

Figure S3: ^{1}H proton NMR of acid digested ZIF-8OD samples

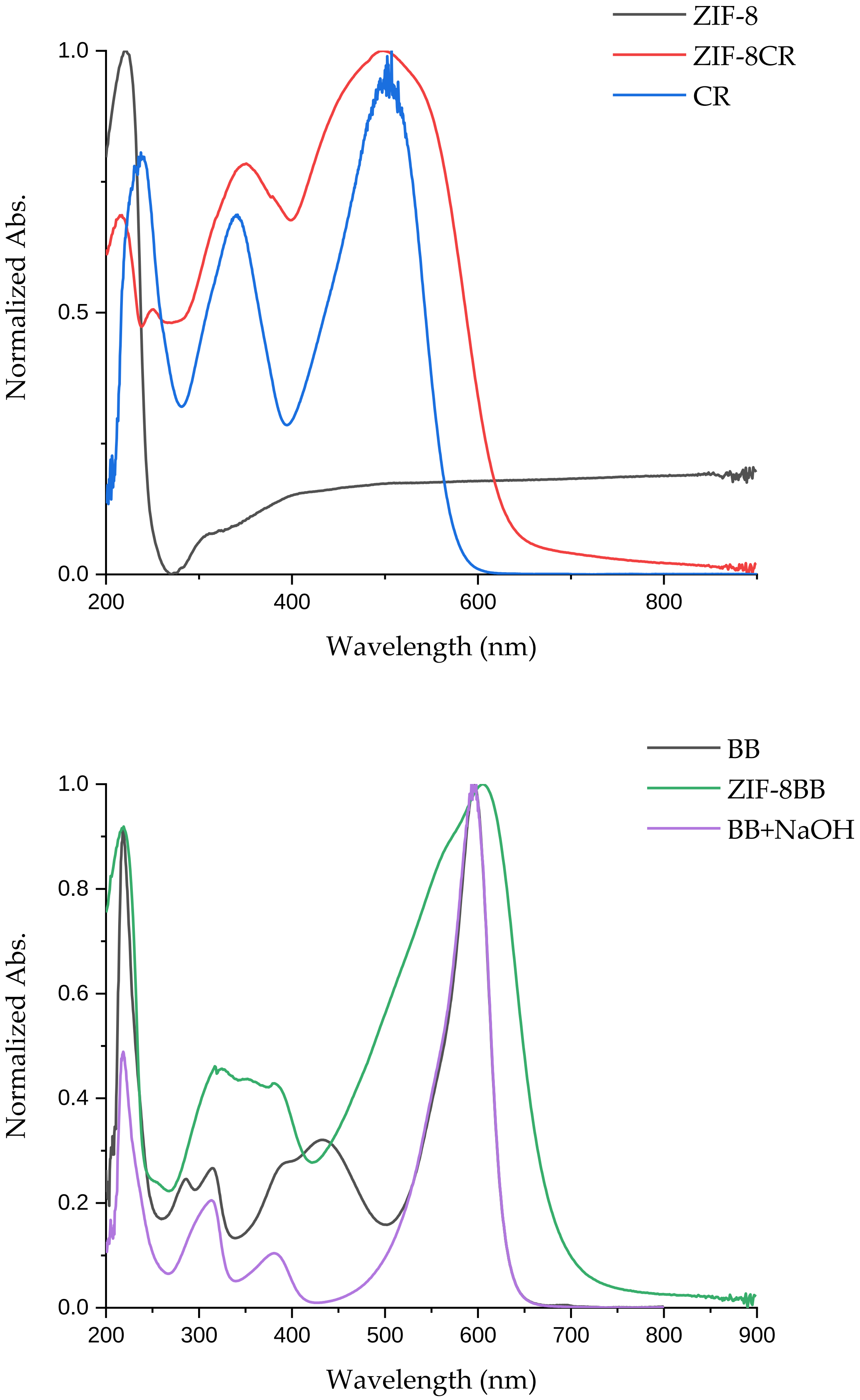
ZIF-8
ZIF-8CR
CR
Normalized Abs.
Wavelength (nm)
1.0
0.5
0.0
200
400
600
800
BB
ZIF-8BB
BB+NaOH
Normalized Abs.
Wavelength (nm)
1.0
0.8
0.6
0.4
0.2
0.0
200
300
400
500
600
700
800
900

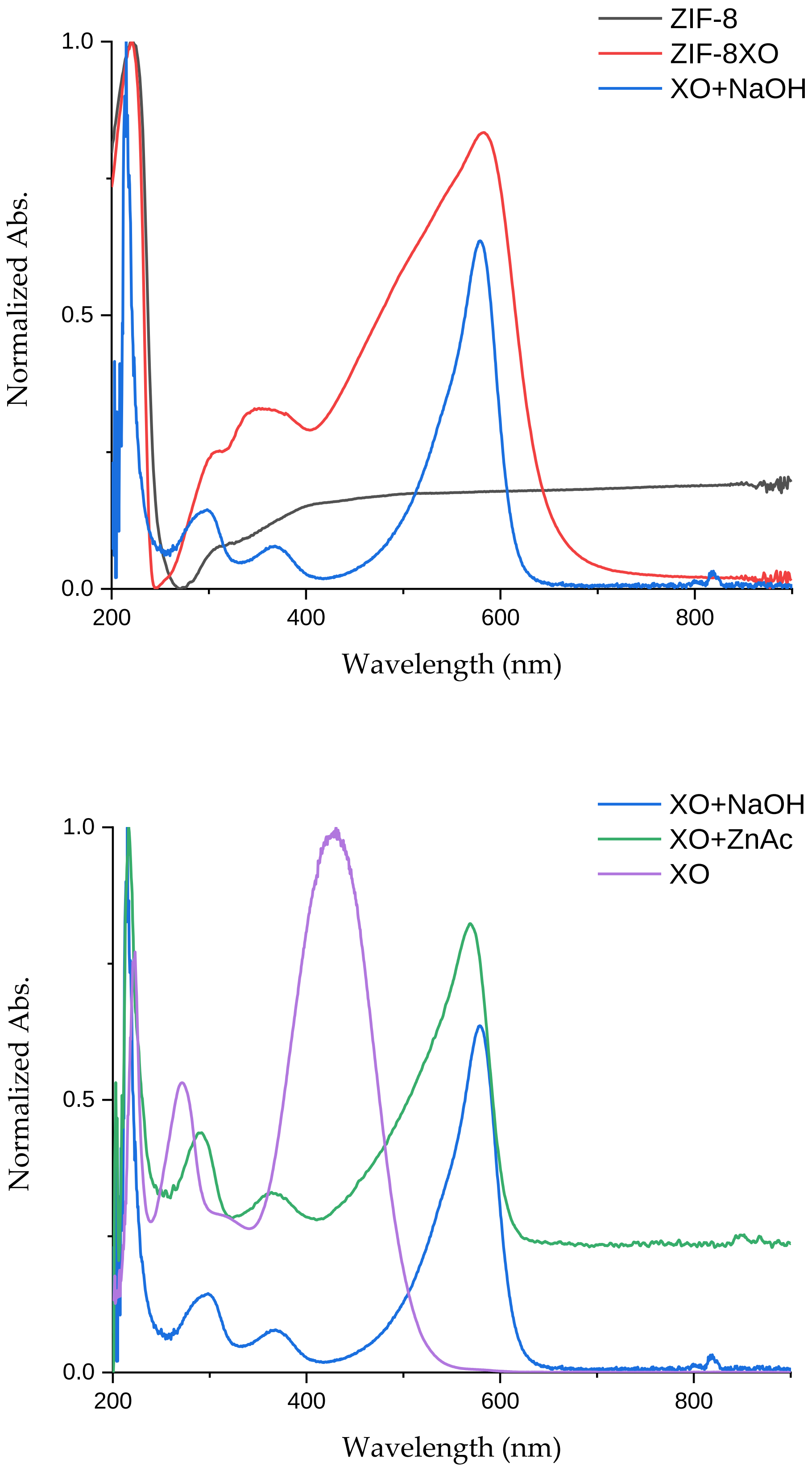

Figure S4: UV-VIS spectra of ZIF-8OD and solutions of OD, spectra of XO and BB solutions with additions of NaOH and zinc acetate were also recorded.

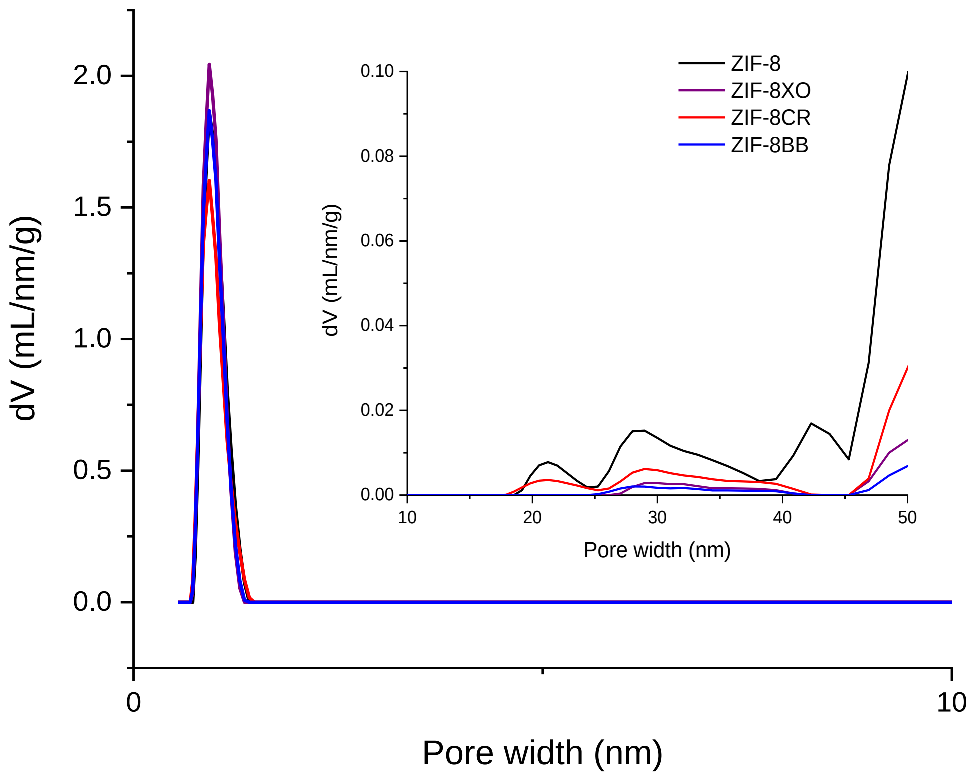

Figure S5: Pore size distribution determined with DFT from nitrogen adsorption data

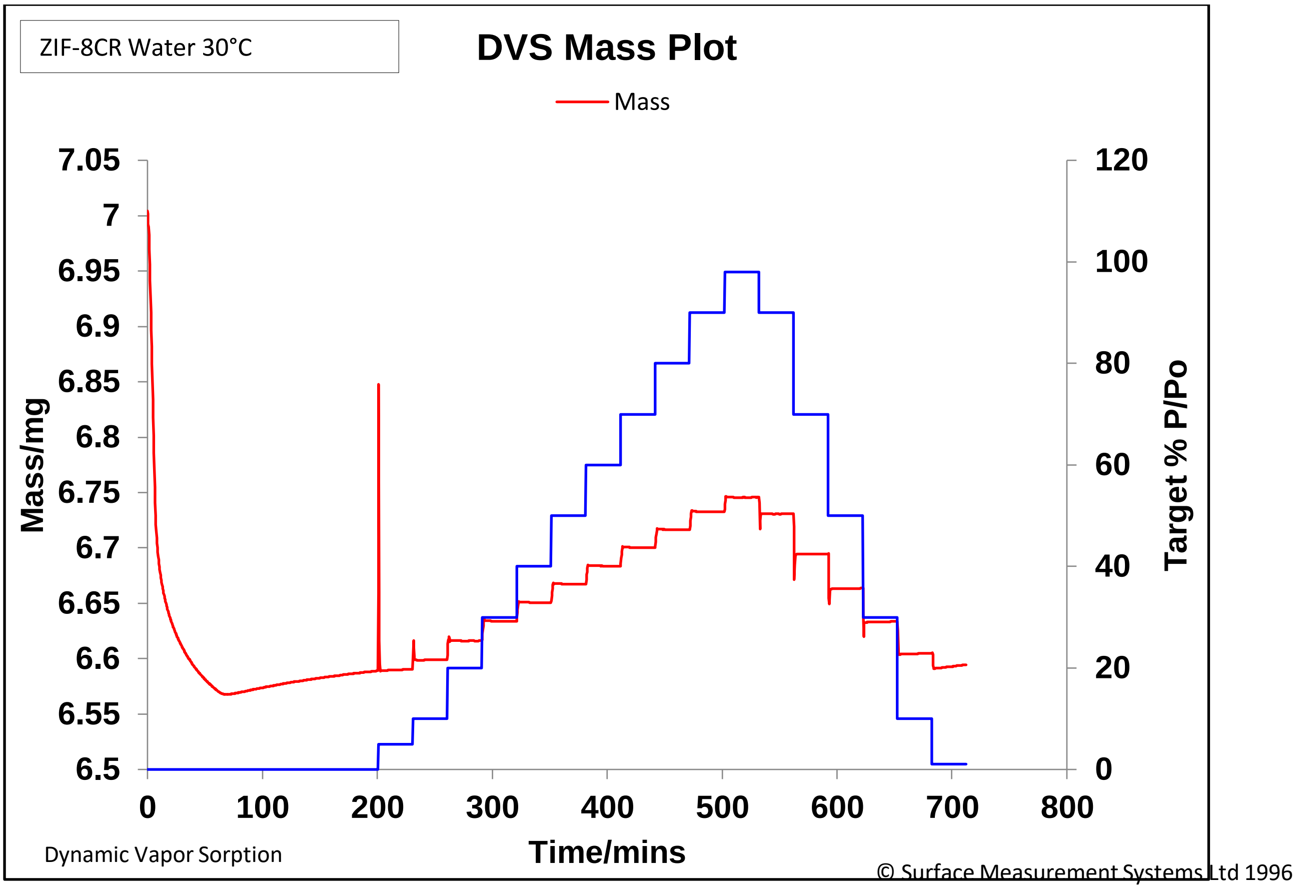
ZIF-8CR Water 30°C
DVS Mass Plot
Mass
7.05
7
6.95
6.9
6.85
6.8
6.75
6.7
6.65
6.6
6.55
6.5
Mass/mg
120
100
80
60
40
20
0
Target % P/Po
0
100
200
300
400
500
600
700
800
Time/mins
Dynamic Vapor Sorption
© Surface Measurement Systems Ltd 1996

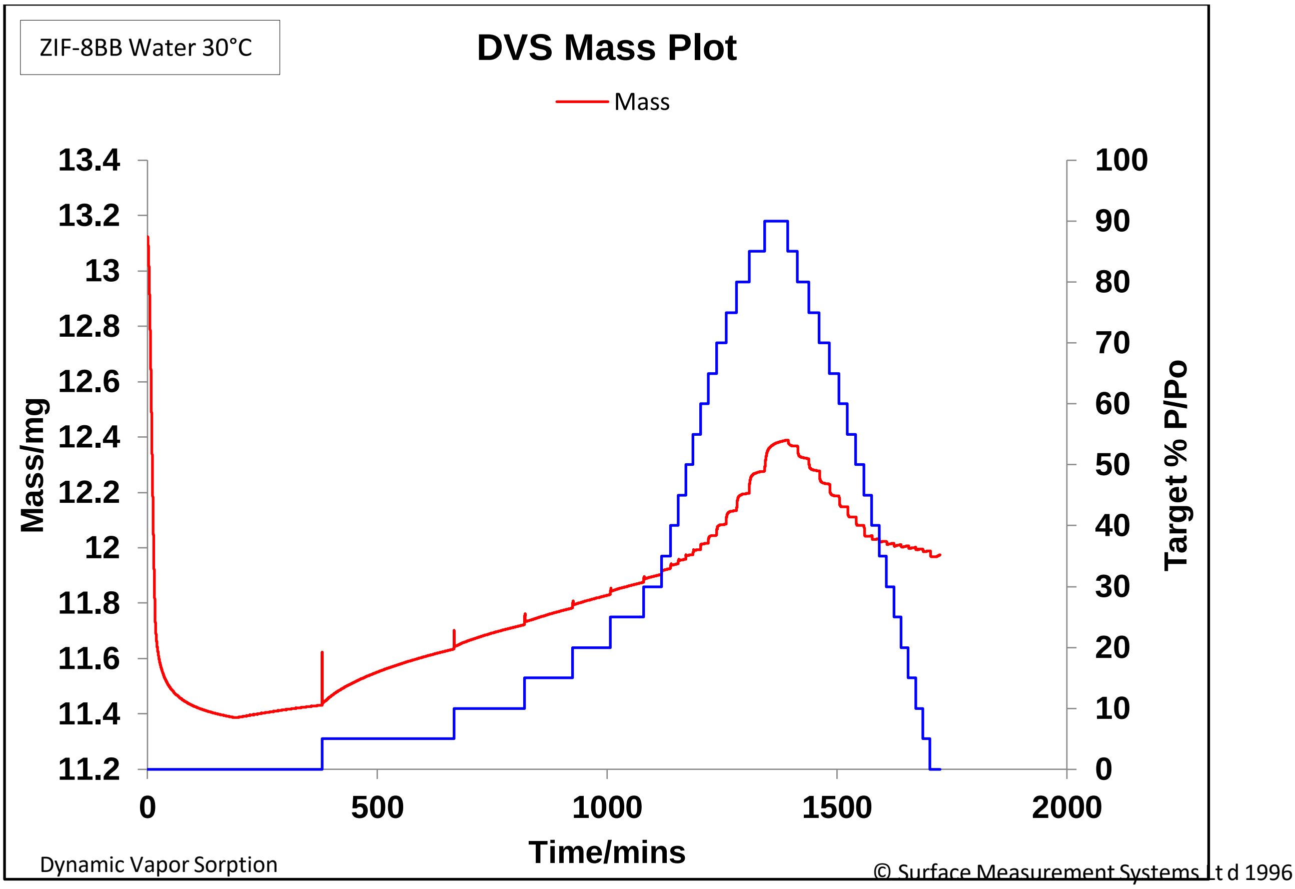
ZIF-8BB Water 30°C
DVS Mass Plot
Mass
13.4
13.2
13
12.8
12.6
12.4
12.2
12
11.8
11.6
11.4
11.2
Mass/mg
100
90
80
70
60
50
40
30
20
10
0
Target % P/Po
0
500
1000
1500
2000
Time/mins
Dynamic Vapor Sorption
© Surface Measurement Systems Ltd 1996

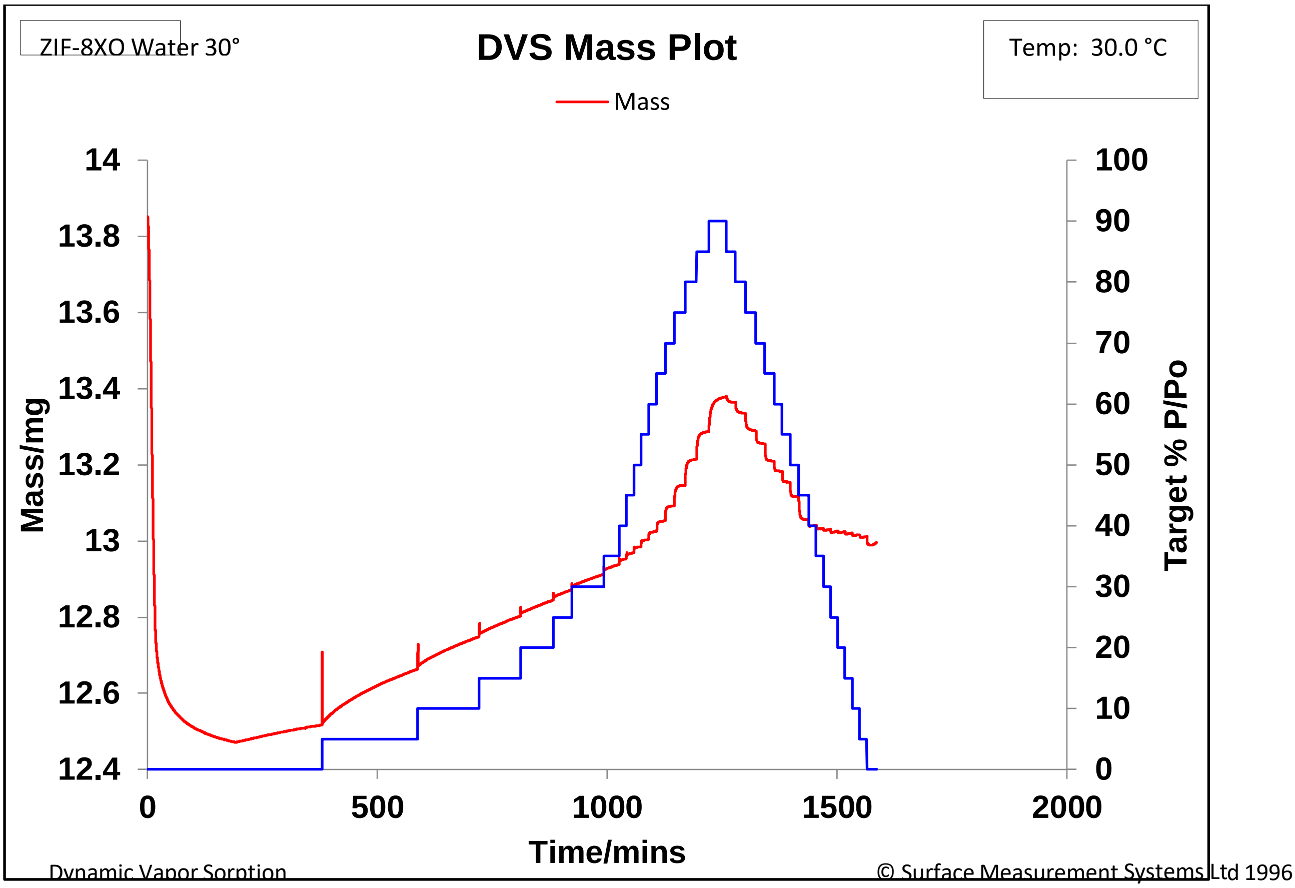

Figure S6: Mass/Pressure time plots for water adsorption of ZIF-8OD (blue line pressure, red line mass)
